# Facility-imposed barriers to early utilization of focused antenatal care services in Mangochi District, Malawi – a mixed methods assessment

**DOI:** 10.1186/s12884-017-1631-y

**Published:** 2017-12-29

**Authors:** Kondwani Chidzammbuyo Mamba, Adamson S. Muula, William Stones

**Affiliations:** 1 District Health Office, P.O. Box 42, Mangochi, Malawi; 2 Department of Public Health, School of Public Health and Family Medicine, College of Medicine, Private Bag 360, Chichiri, Blantyre, 3 Malawi; 3 St George’s University of London, SW17 ORE, London, UK

**Keywords:** Focused antenatal care, Health promotion, Male involvement, Universal access

## Abstract

**Background:**

Focused Antenatal Care (FANC) is advocated by the World Health Organization (WHO) as a key service approach to improving the health of pregnant women. Four targeted visits to antenatal clinics are recommended starting in the first trimester. First trimester attendance for FANC in Mangochi District, Malawi was low at 8%. FANC has mainly been promoted through health facility based communication activities with less emphasis on activities at community level. We developed and tested a community focused health communication approach “Community Driven Total FANC Attendance (CDTFA)” with the aim of increasing FANC clinic attendance. We included a research component in order to understand the context and responses of community members to this intervention.

**Methods:**

CDTFA meetings were designed in parallel with data gathering with approval of the local research ethics committee and community stakeholders. Participants in both the CDTFA meetings and data gathering activities, undertaken from December, 2015 to June, 2016 were of reproductive age (15–49 years). Data were collected through flexible interactive processes from participants through recording on pre-designed forms. Quantitative data were processed and analyzed in Microsoft Excel, while qualitative data were manually analyzed to identify themes.

**Results:**

In total, 403 CDTFA meetings were held. In the course of interactions with community members, some barriers that affected early utilization of FANC services were identified. Women who did not bring their partners and those who could not bring along with them cloth wraps for the newborn to clinics were not allowed to access FANC services. Payment for authorization letters from village heads for women who have no partners and user fees in non-governmental health facilities were also identified as barriers.

**Conclusions:**

Despite the benefits of FANC services, health authorities in the District should ensure that use and promotion of the approach does not inadvertently bar some pregnant women from accessing services. There is a need to explore strategies and redesign an approach to health promotion that will promote uptake of the integrated services in FANC clinics without infringing on women’s rights to access health care.

**Electronic supplementary material:**

The online version of this article (10.1186/s12884-017-1631-y) contains supplementary material, which is available to authorized users.

## Background

According to World Health Organization (WHO) estimates, about 303,000 maternal deaths occurred in the world in 2015. Of these deaths, 66% occurred in sub-Saharan Africa followed by Southern Asia at 22%. About 99% of the maternal deaths occurred in low and middle income countries and most could have been prevented. The primary causes of maternal deaths are haemorrhage, hypertensive diseases of pregnancy and sepsis, and indirect causes, mostly due to interaction between pre-existing medical conditions and pregnancy [1].

Focused antenatal care (FANC) provides a package of services that contributes to the health and wellbeing of a woman throughout her pregnancy, childbirth and postnatal period. Implementation of FANC was adopted by the WHO in 2002, replacing the traditional antenatal care (ANC) service model. In the FANC model, women are classified into either basic group (those with low risk pregnancies), or specialized group (those with high risk pregnancies) basing on the existing health risks identified on the women’s first clinic visit. Women may therefore change their groups (whether basic or specialized) in subsequent visits depending on the absence or availability of pregnancy related risks and complications or changing clinical conditions. For routine FANC, four targeted antenatal clinic visits are recommended with the first visit occurring in the first trimester of pregnancy (between 8 and 12 weeks). The number of visits for women with specific conditions are more than four depending on their case needs related to their condition. In order to realize the full benefits that FANC can offer, there is a need for all pregnant women to attend FANC clinics for at least each of the four visits [2, 3].

Significant variations of FANC clinic attendance by women in the first trimester have however, been observed across regions with the lowest proportion being noted in the sub-Saharan Africa region. Recent reports on FANC utilization in the first trimester gleaned from different Demographic and Health Surveys (DHS) across African countries such as Ethiopia, Congo-Brazzaville, Ghana, Zambia, Tanzania and Malawi indicate that fewer than 30% of pregnant women attend in the first trimester [4–9].

Many countries in sub-Saharan Africa are stepping up efforts to encourage pregnant women to utilize FANC services. Community based health promotion programmes that aim at mobilizing women to utilize services are being undertaken using different approaches depending on local circumstances. One such intervention is to encourage pregnant women to attend FANC clinics with their male partners, to address the context of health care decisions mostly being made by men and mothers in-law [10]. The dominance of men in society puts pregnant women at risk because they usually do not have adequate knowledge of reproductive health issues and their decisions may not adequately embrace the elements of optimal sexual and reproductive health services [11].

In Malawi, the national coverage of first trimester FANC services is low at 12% [12]. Recently, WHO has extended recommendations for more comprehensive coverage of antenatal interventions with up to eight contacts for additional components of care, but in Malawi and many sub-Saharan Africa countries, the challenge remains to achieve the earlier standard of four FANC visits [13].

The national context of low FANC clinic attendance is not different in Mangochi District and has been oscillating between 6 and 10%. About 90% of pregnant women in the District are therefore denied early initiation of essential services such as early screening tests for HIV, STI and malaria and identification of obstetric complications such as pre-existing hypertension and anaemia that are normally offered by health facilities in the District [14, 15]. Barriers were long distance to the health facility, fear of wizards and witches affecting the pregnancy if it became known, and a cultural norm requiring women to seek permission from their husband before attending clinics in rural Malawi [16].

Through the Safe Motherhood Initiative, Malawi is using a community engagement approach to increase health facility-based delivery and FANC clinic utilization by pregnant women [17]. The District Health Office (DHO) in Mangochi is involving community leaders to bringing about positive changes related to safe motherhood. Community leaders and Health Centre Management Teams have enacted by-laws with socially acceptable local fines for people who do not comply with the edicts of the by-laws. For example, in one area in Mangochi, there is a fine of a goat if a pregnant woman delivers at home.

There were no active community based health communication elements observed promoting FANC attendance to complement efforts made at health facilities. In order to address this gap, a community based health communication strategy called Community Driven Total FANC Attendance (CDTFA) was developed and implemented in some communities in the District. In the course of implementing this approach, we conducted a study aiming at identifying barriers that were inadvertently working against the objective of increasing FANC attendance in the first trimester.

## Methods

**Fig. 1:**
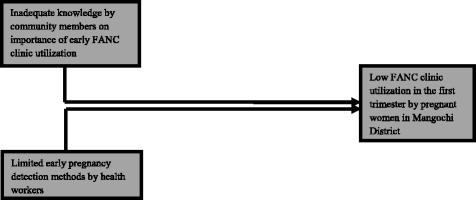
Factors contributing to low FANC utilization in the first trimester by pregnant women in Mangochi District

This is a cross-sectional study that collecting qualitative and quantitative data from both communities through participants during village level meetings and health facilities through registers and interviews with pregnant women. Inadequate knowledge among community members about the importance of early FANC clinic utilization was assumed by health workers during the 2013/2014 Health Management Information System (HMIS) and District Implementation Plan (DIP) review meeting to be one of the factors contributing to low FANC clinic utilization in the first trimester by pregnant women in the District. Figure 1 illustrates some contributing factors to low FANC clinic utilization in the District.

### The setting and implementation of the CDTFA approach

The District has a total population of 1,017,070 of which 528, 876 are females. The majority of people speak and understand the vernacular Chichewa language while English is an official language. The majority of the population have low income levels and live with less than one US Dollar ($) per day. The District has 42 health facilities consisting of four hospitals, 28 health centers and 10 dispensaries. Of the 42 health facilities, 25 are government owned and 17 are privately owned. Among the private health facilities, 15 are owned by the Christian Health Association of Malawi (CHAM) and two by private practitioners. These health facilities cater for a total of 1254 villages in the District which are administratively grouped into 12 Traditional Authority areas [18]. This study was conducted in 403 villages under 19 health facilities in the District, and was implemented under the authority of the District Council. The study team consisted of the Principal Investigator (PI), district based health workers – two clinicians, three nurses, two Assistant Environmental Health Officers (AEHO) and two Senior Health Surveillance Assistants (SHSA) as master trainers; health facility based health workers in total consisting of – 19 Medical Assistants, 19 nurses, five AEHO, 19 SHSA and 146 Health Surveillance Assistants (HSA) as facilitators.

Before implementation of the approach, the PI conducted a 3 day training of ‘master trainers’ who were trained on CDTFA meeting facilitation and triggering, supervision, and data collection. The master trainers in turn conducted a series of 2 day facility based trainings for village level CDTFA facilitators. Village level meetings and interviews were conducted by facilitators from the health facility of the same catchment area.

All data collection tools for meetings and interviews were translated from English to Chichewa by two Secondary School language teachers in the District. Both master trainers and facilitators were fluent in the Chichewa language. Participants were men and women of reproductive age (15–49 years), community leaders and older women (who have active community roles in matters related to reproductive health) who attended the CDTFA meetings. Women were asked to bring with them to the meetings their FANC health profile records booklets known as “Health Passports”. Community members were also facilitated to choose volunteers called “lead mothers” who were assigned roles of identifying and encouraging pregnant women to start FANC clinics in the first trimester.

### Data collection

All data collection for assessment of the intervention was done alongside meetings from December, 2015 to January, 2016. Qualitative data were collected during the meetings through flexible interactive processes from a sample involving all men and women who attended village level CDTFA triggering meetings. While the meeting was in progress, facilitators gathered health profile records and noted those who had or had not started antenatal care in the first trimester in their most recent pregnancy (Additional file 1). The facilitators later asked these women to give open responses on reasons for either early or late attendance. In addition to tape recording, facilitators recorded the explanations that were given by women on pre-designed data collection forms. These forms included information on the day of the meeting, names of facilitators, attendance of the 15–49 year population at the event, the number of health profile books collected and scrutinized by facilitators, the number of first trimester attendees identified, brief notes on reasons for starting clinic attendance either early or late, and details of “lead mothers” selected by the community (Additional file 2).

Six months later, in June, 2016 as a preparation for a second round of CDTFA implementation, further data collection was undertaken. During this time, health centre FANC registers were used to identify women who initiated FANC either early or late during the preceding 6 months period. The identified names were sorted according to the women’s respective villages and were submitted to the responsible HSA who then interviewed them using a pre-designed open response questionnaire in order to identify main reasons why they had attended FANC either early or late. The interviews used pre-designed forms to record responses from the women (Additional file 3).

The second CDTFA meetings were held in all the study villages soon after these interviews. The issues that were identified during interviews with the pregnant women were communicated back to their respective communities as part of feedback during the follow up village CDTFA meetings. This feedback was aimed at creating community awareness on issues associated with FANC clinic attendance at village level so that local solutions could be suggested in order to increase FANC clinic utilization in their respective localities.

### Data analysis

Individual participant data collected during the CDTFA meetings and interviews were anonymized for analysis, presentation and community feedback. All completed village level CDTFA meeting data collection forms were consolidated at health centers to come up with a CDTFA activity report (Additional file 4). The PI consolidated the 19 health centre field notes reports, recorded audios and filled questionnaires for analysis. Recorded audios were transcribed and translated into English. Analysis was done by inductive processes driven by the data itself. The PI familiarized himself with the data through reading and re-reading to look for patterns and important issues. The PI then manually identified and sorted related codes into meaningful groups to come up with categories. The categories were further collapsed to form initial themes. These initial themes were further refined and defined from which interpretations of the data regarding barriers to early clinic utilization were based on [19]. For triangulation purposes, these processes were also repeated by a District based qualitative researcher.

Quantitative data from structured interviews were entered into Microsoft Excel. Through reading and re-reading of responses, categories of reasons for early and late initiation of FANC clinic by pregnant women were identified. All related responses were then grouped under these categories. After the grouping of responses, frequencies of responses under each category were tabulated. The identified reasons provided village level specific issues which were used as feedback to inform CDTFA facilitators about the type of health communication messages to be tackled during subsequent CDTFA meetings.

## Results

A total of 403 CDTFA trigger meetings were conducted in each of the villages under the 19 health centers of the District. Out of a total population of 204,532 men and women of childbearing age in the villages, 49,797 (25%) attended the first CDTFA meetings. The percentage of women of childbearing age in the villages who attended the CDTFA meetings was 39%. Among the participants who attended the CDTFA meetings, the proportion of women was however, higher at 83% than that of men at 17%. Out of the 41,644 FANC health records booklets that were collected and checked by facilitators during the meetings, only 1548 (mean 4%; range 1–8) had first FANC clinic visit in the first trimester.

During follow up of the health facility registers 6 months later, a total of 3147 pregnant women were identified. Of these, 1015 (32%) women had started their FANC clinics in the first trimester. Table 1 shows the reasons the women gave for starting FANC early or late ranked according to frequencies.

**Table 1:** Reasons^a^ given by women for starting FANC clinic early or late

Reasons for early FANC clinic start [Number of women who mentioned the reason (%)] *N* = 1015	Reasons for late FANC clinic start [Number of women who mentioned the reason (%)] *N* = 2132
1. Had knowledge on importance ANC =477 (47)	1. Husband not available =362 (17)
2. Adherence to health messages/advice from health workers =142 (14)	2. No money to pay for FANC services =298 (14)
3. Went there because of sickness =122 (12)	3. Own choice =298 (14)
4. To know if I was pregnant = 92 (9)	4. Unaware of pregnancy =277 (13)
5. Encouraged by close relatives/husband =51 (5)	5. No means of transport =256 (12)
6. Fear to pay by-law fines =41 (4)	6. No cloth wraps for the baby to show at FANC =192 (9)
7. To be well received by nurses during delivery =30 (3)	7. Lack of knowledge on importance of FANC = 107 (5)
8. Encouraged by local leaders =30 (3)	8. Long distance to FANC clinics =107 (5)
9. No reason given =30 (3)	9. To reduce FANC visits =85 (4)
	10. Waiting for visible signs of pregnancy = 64 (3)
	11. Was too sick to go for FANC = 47 (2)
	12. Cultural beliefs = 21 (1)
	13. Was outside the country = 21 (1)

^a^Each woman was asked to provide one main reason

### Identified barriers to utilization of FANC in the district

Four main themes were identified as barriers.

1. “A pregnant woman can access FANC services only if she has a spouse to accompany her to the clinic”
  It was reported during CDTFA meeting discussions in most health facilities that there are by-laws formulated by health officials and community leaders requiring that every pregnant woman is to be accompanied by a spouse when going to attend FANC clinics. In situations where a spouse is not available, a pregnant woman is supposed to bring with her a stamped letter from her village head attesting the unavailability of a spouse. During the CDTFA meetings, it was reported that for most women to get the letter of exemption from a village head a monetary fee had to be paid. Various amounts of fees ranging from the equivalent of 1.5–4 US$ were reported in different areas. When the women were asked why they started attending FANC clinics late, 17% (*n* = 362) indicated the unavailability of their spouses to escort them to the health facility as per stipulations of the by-laws. One woman explained this as follows:
*“You cannot be assisted by doctors if you go to the hospital without a spouse. I did not go to ANC clinic early because my husband was in South Africa. I could not afford to pay for the letter from the village head”.* – a female participant.
2. “Male involvement in reproductive issues contradicts their traditional norms”
  Another reason reported during the CDTFA meetings for women not to bring along their spouses is that according to tradition, issues related to pregnancy and childbearing are mostly viewed as women’s responsibilities. Men therefore, do not want to go with their spouses to FANC clinics for fear of being laughed at and stigmatized by their fellow men.
*“Our nurses should just be accepting our women to access ANC services even if they go alone. Men in this village feel shy to accompany their spouses to clinics. Starting from parents, pregnancy related issues have always been handled by women”.* – male participant.
3. “Men view FANC clinics as platforms for forcing them to be tested for HIV”
  Following the national policy guidelines, PMTCT is being implemented in FANC clinics in all health facilities of the District where couple counseling and non-coercive HIV testing are promoted. Community members fear the associated stigma and discrimination should they test positive as illustrated in the quote below:
*“Is it possible to stop coercing male partners getting an HIV test when they accompany their spouses to clinics? I see that male partners do not want to accompany their spouses because they do not want to be tested for HIV”* – a female participant.
4. “FANC services are for wealthy people”
  At community level, women are encouraged to save money and supplies to be used during delivery. During CDTFA meetings, it was reported that as part of this preparedness, health workers demand that pregnant women bring with them cloth wraps for the baby (traditional *‘chitenje’* singular/ *‘zitenje’* plural) at their initial FANC visit. The number of *‘zitenje’* requested ranges from one to four and this demand was viewed by 9% (*n* = 192) of pregnant women who started FANC clinics late as unaffordable and contributed to their delay to start FANC clinics.
*“If my wife tells me that she is pregnant, it becomes difficult for me to source four ‘zitenje’ within three months of her pregnancy as demanded from the nurse at the hospital. In this case, I tell my wife to wait until I find money to buy the four ‘zitenje’. I feel the demand is too much for poor people like us.”* - a male participant.
  The cost of one *‘chitenje’* locally was reported to be the equivalent to 1.5 US$. Participants in CDTFA meetings stated that they could not afford the total cost of four cloths within the first 3 months of pregnancy as required by the health facilities. Due to this requirement for birth preparedness, some pregnant women especially those who did not have spouses reported it caused them to incur additional costs for them to access FANC services. If a pregnant woman did not have a spouse, or the spouse was not available during her time to start FANC clinics, she in addition to sourcing a letter from the village head, had to meet the cost of *‘zitenje’*.
*“We have a by-law that was enacted by the chiefs in this area after receiving concerns from the health centre that most men were not accompanying their spouses to ANC clinics. We do not punish pregnant women who do not have spouses. We advise them to go to their village head to collect letters for them to be accepted at the health centre. The fee that she is complaining of is not meant for such pregnant women only. The fee is for any stamped letter that everyone pays when one asks for it from the chief”* – a village head*.*
  While FANC health services are free at the point of use in government facilities, communities living in areas under CHAM health facilities pay user fees. Integration of services in FANC clinics means their workload has increased in terms of consultation and medical supplies. Pregnant women in these areas have to pay for FANC clinic services. During the CDTFA meetings, most women who attended FANC clinics in CHAM facilities attributed their late initiation to the user fees as evidenced by the following quote:
*“The services are not free at our health facility. We pay 2,000 Kwacha* [3 US$] *to access services which many of us do not afford. Is it possible for government to be paying for us money for ANC so that we can be accessing ANC services free of charge as is done in government facilities?”* – a community leader from an area under a CHAM health facility.

## Discussion

Our intervention reached approximately 39% (41,477 women) of those of childbearing age. The low level of male participation (17%) in reproductive health related activities has also been reported in many sub-Saharan Africa countries contexts including Tanzania, Kenya and Cameroon [11, 20, 21].

Six months after implementation of CDTFA trigger meetings, community based assessment of first trimester attendance at clinics showed an increase from 4 to 32%. This increase cannot however, wholly be attributed to implementation of CDTFA meetings. During the study period other interventions were deployed at some sites including urine pregnancy-testing in line with the framework shown in Fig. 1. Despite interventions, the percentage of women with delayed first attendance was still substantial at 68%. Promotion messages for men’s participation in FANC clinics, routine HIV testing for couples during FANC clinics, and a requirement for pregnant women to bring cloth wraps as proof for birth preparedness, though with good intentions, have inadvertently deterred some women from accessing FANC services in the first trimester. In addition, the payment of user fees in CHAM and private health facilities has also been shown to bar pregnant women from accessing essential FANC services.

Three explanations came out of the CDTFA meetings why male partners do not escort their spouses to FANC clinics. Firstly, some men in Mangochi District live for significant amounts of time away from their homes because they mainly rely on small business, some men leave their families for neighbouring Mozambique and South Africa for employment opportunities [18]. Secondly, low male involvement in the District is being linked to culture that view issues of pregnancy and childbirth as female responsibilities. Thirdly, HIV testing services being done in FANC clinics are also discouraging participation of men because of fear of stigma and discrimination. These findings are consistent with those from Kenya [20]. Globally, there has been a drive to increase male involvement in reproductive health services including FANC. Studies across the sub-Saharan Africa region have shown that increasing partner involvement can influence utilization decisions by women of reproductive services like HIV testing and PMTCT measures [11, 22, 23]. Furthermore, there is evidence that involvement of men has an effect of lowering attrition rates in FANC and PMTCT programmes [10, 24].

While appreciating the need for pregnant women to receive FANC services with their partners, the health authorities in the District need to identify other strategies to increase partner involvement rather than the use of by-laws. In a Tanzanian study, systematic coaching of pregnant women on how to invite their partners followed by written invitations resulted in an increase in male partner participation in FANC services [25]. In a similar study on women’s attitudes towards male involvement in FANC in Lesotho, improvement in structural design and clinic services and a comprehensive community mobilization on male involvement were recommended to increase partner involvement in FANC services [26].

Poverty is another barrier to utilization of FANC services by communities in the District. About 73% of people in Mangochi are classified as poor and live below the World Bank prescribed 1 US$ per day threshold [27]. Despite this economic status, utilization of FANC services by pregnant women in Mangochi District is being associated with some financial costs. Mangochi District Health Office is implementing a Service Level Agreement (SLA) with CHAM and private practitioners in maternal and neonatal health services. In these contracts, the Ministry of Health through the District Health Office pays for maternal and neonatal health bills that are incurred by women in areas where there are no government health facilities. The contracts however, mostly cover costs at delivery and the neonatal period and do not extend to FANC services. A review of these contracts may be necessary to determine whether it is feasible to include FANC services in the agreements. Findings about the costs associated with FANC clinic attendance in Mangochi District are consistent with observations from a study Ghana, Kenya, Malawi and Nigeria [28, 29]. Socio-economic status of women and their husbands was related to the timing of initiation of FANC clinics.

From a rights based perspective it can be argued whether these prescriptive requirements are fair. The financial and logistical challenges faced by pregnant women in our District cannot easily be met through women’s own resources. Universal access to health care implicates elimination of geographical, economic, social-cultural, and organizational or gender barriers that impede all people from using integral health services [30].

The findings presented here reflect experience gained working with communities and were largely un-anticipated: we had not specifically intended to collect and analyze data on service related barriers in our CDTFA implementation. It is the richness of the views and explanations that were associated with timing of starting FANC clinics from community members during the initial stages of introducing CDTFA meetings that ignited our interest to collect and analyze the data presented in this paper. Taking into consideration our local context, the findings of this study are in consistent with experience from Rwanda, Nigeria and Tanzania [2, 11, 15].

### Strengths and limitations

The participatory facilitation of the meetings allowed participants to freely give their opinions which were further discussed by all. At the end of each meeting, facilitators gave summaries to enable consensus. The separate interviews that were conducted with women provided an additional opportunity for participants to share views. This was implementation research so we considered it appropriate to use data collectors affiliated to health centers serving the same catchment areas. It is possible that additional barriers could have been identified if external data collectors had been deployed who may have been perceived as more independent.

## Conclusions

In our setting, implementation of an internationally mandated policy has been associated with unintended barriers constraining improvement in FANC utilization. The first visit prescriptive requirements (partner involvement and cloth wraps for maternity) were reported to bar pregnant women from accessing FANC services. In addition, the fees that were being imposed on women who do not have a partner to accompany them and user fees at private health facilities were not affordable for poor pregnant women in the District. Consequently, a significant proportion of women in the District are unable to access services in the first trimester, with consequent risk of adverse outcomes. Nationally mandated programming needs to address local implementation elements in order to realize the full gains in access to antenatal services and avoid inadvertently institutionalizing barriers to access. There is need for the District authorities to explore strategies that will promote uptake of integrated services in FANC clinics without infringing women’s rights to access health services. District based stakeholder engagement can help to redefine some of the community mobilization strategies to enable community members to come forward, express their needs and challenges, and engage in genuine partnership with service providers. Policies on free access to maternal health services also need to be reinforced to remove indirect burdens of the type identified here.

## Additional files

Additional file 1: CDTFA meeting facilitation guide. (DOCX 16 kb)

Additional file 2: Village meeting data collection form. (DOCX 12 kb)

Additional file 3: Interview guide for pregnant women. (DOCX 17 kb)

Additional file 4: Health centre village meetings summary form. (DOCX 12 kb)

## Abbreviations

ADDRF: African Doctoral Dissertation Research Fellowship
ANC: Antenatal Care
CDTFA: Community Driven Total FANC Attendance
CHAM: Christian Health Association of Malawi
DHO: District Health Officer
DHS: Demographic and Health Survey
DIP: District Implementation Plan
FANC: Focused Antenatal Care
HIV: Human Immuno Deficiency Virus
HMIS: Health Management Information System
HSA: Health Surveillance Assistant
ICEIDA: Icelandic International Development Agency
PMTCT: Prevention of Mother to Child Transmission
US$: United States Dollar
WHO: World Health Organization
