# Supplementary material for: Facility-imposed barriers to early utilization of focused antenatal care services in Mangochi District, Malawi – a mixed methods assessment
Source: BMC Pregnancy Childbirth. 2017 Dec 29;17:444. doi: 10.1186/s12884-017-1631-y (PMC5747179; doi:10.1186/s12884-017-1631-y)
Supplement: Supplementary file 1 — CDTFA meeting facilitation guide. (DOCX 16 kb) [file 12884_2017_1631_MOESM1_ESM.docx]

**Additional file 1. Community Driven Total Focused Antenatal Care village level meeting facilitation guide**

| **SN** | **STEP** | **ACTIVITY** |
| --- | --- | --- |
| 1 | Getting the meeting started | The Health Surveillance Assistant (HSA) the convenor of the meeting facilitates the opening prayer and introductions for meeting participants |
| 2 | Welcoming remarks | The convenor invites the Village head to give welcoming remarks |
| 3 | Meeting objective | The convenor invites the facilitator to give the objective of the meeting to participants |
| 4 | Gathering of health profile records books | Convenor invites facilitators to collect health profile records books from women |
| 5 | Discussion on Focused Antenatal Care (FANC) | Convenor invites the facilitator to moderate a discussion on FANC including definition of FANC, services offered under FANC, schedules of FANC, importance of FANC services and importance of early FANC utilization. Meeting participants to be given adequate time to discuss on specific FANC topics. Later, the facilitator to summarizes by reaching agreement with participants on areas where they gave correct information and making corrections on areas where communities did not give the right information. |
| 6 | Identification of First Trimester Attendees | While a discussion on FANC is in progress, facilitators sorts out the gathered health profile records books to identify women who attended FANC clinics in the first trimester during their recent pregnancy. |
| 7 | Announcement of First Trimester Attendees | Convenor invites facilitator to announce names of those who attended FANC clinics in the first trimester during their recent pregnancy. |
| 8 | Recognition of First Trimester Attendees | Convenor invites the identified first trimester attendees to come in front. The convenor further invites the village head and other opinion leaders to shake a hand of recognition to the first trimester attendees and have a photograph together. |
| 9 | Identification of possible reasons for early FANC attendance | First the facilitator to seek views from first trimester attendees on what made them start FANC clinics early. The facilitator then asks the general meeting participants of the meeting on what would make a pregnant woman start FANC clinics early in their area. |
| 10 | Identification of possible reasons for early FANC attendance | Firstly, the facilitator to seek views from volunteers amongst the women who started FANC clinics late. Secondly, the facilitator to ask the general meeting participants of the meeting on what would make a pregnant woman start FANC clinics late in their area. |
| 11 | Formulation of village action plan | Facilitator leads a discussion on actions to be taken by community members to make sure that pregnant women start FANC clinic services early. |
| 12 | Nomination of Lead Mothers | Facilitator discusses with the community on the need to have some women to lead in the implementation of the action plan and to encourage pregnant women to start FANC clinics early. She/He then facilitates the nomination of three women called 'Lead Mothers' |
| 13 | Closing remarks | Convenor invites the village head to deliver closing remarks |
| 14 | Meeting close up | Convenor asks a volunteer to give a closing prayer |
| 15 | Distribution of health profile records books back to owners | Convenor and facilitators distributes the collected health profile records books back to owners |
