# Supplementary material for: Facility-imposed barriers to early utilization of focused antenatal care services in Mangochi District, Malawi – a mixed methods assessment
Source: BMC Pregnancy Childbirth. 2017 Dec 29;17:444. doi: 10.1186/s12884-017-1631-y (PMC5747179; doi:10.1186/s12884-017-1631-y)
Supplement: Supplementary file 3 — Interview guide for pregnant women. (DOCX 17 kb) [file 12884_2017_1631_MOESM3_ESM.docx]

**Additional file 3. Interview guide for pregnant women who started Focused Antenatal Care clinic from the month of January to June 2016.**

**Instructions:**

1. Greet the participant before you start interviewing and thank her for accepting to participate in the study
2. Give the participant the consent form to read or read it to her before you start the interview
3. Ask for ANC health profile records book
4. Ask the participant to sign the consent form to show agreement to participate in the study
5. After the interview thank the participant for participating in the study.

| **Section 1: Social Economic and Demographic Characteristics** | | | |
| --- | --- | --- | --- |
| **Question number** | **Question** | **Options** | **Skip** |
| 1 | Participant ID |  |  |
| 2 | Date | / / |  |
| 3 | Age |  |  |
| 4 | Health Facility Code |  |  |
| 5 | Marital Status | Single……………………………………1  Married………………………………….2  Divorced………………………………...3  Widowed………………………………..4 |  |
| 6 | Traditional Authority | Chimwala……………………………….1  Bwananyambi…………………………...2  Chowe……………………………………3  Jalasi……………………………………..4  Katuli…………………………………….5  Makanjira………………………………..6  Nankumba……………………………….7  Namavi…………………………………..8  Mponda………………………………….9  Chilipa…………………………………10 |  |
| 7 | Village Code |  |  |
| 8 | Education Status | None………………………...1  Primary……………………...2  Secondary…………………...3 |  |
| 9 | Religion | Christianity………………….1  Islam………………………...2  Other………………………...3 |  |
| 10 | Occupation Status | Employed……………………1  Unemployed…………………2  Business……………………...3  Other(Specify).………………4 |  |
| 11 | Tribe | Yao…………………………...1  Chewa/Nyanja………………..2  Tumbuka……………………...3  Lomwe………………………..4  Tonga…………………………5  Ngoni………………………….6  Other(Specify)………………...7 |  |
| 12 | Is she a first trimester Attendee? | Yes……………………………………………  No……………………………………………. | If Yes Skip Q 18.  If No Skip Q 17. |
| **Section 2: Knowledge on focused antenatal care** | | | |
| 13 | Can you tell me how you understand antenatal care? |  |  |
| 14 | When do you think is a pregnant woman supposed to start antenatal care? |  |  |
| 15 | Can you mention any benefits for antenatal care |  |  |
| 16 | Can you mention any benefits to starting antenatal care early? |  |  |
| **Section 3: Reasons for starting antenatal care either early or late** | | | |
| 17 | From the health profile records book, we have seen that you started your antenatal care clinic early. Can you give the main reason that made you start early? |  |  |
| 18 | From the health profile records book, we have seen that you started your antenatal care clinic late. Can you give the main reason that made you start late? |  |  |
| **The End**  **Thank you very much for participating in the survey. All the information collected from you will be kept confidential.** | | | |
