# Supplementary material for: Facility-imposed barriers to early utilization of focused antenatal care services in Mangochi District, Malawi – a mixed methods assessment
Source: BMC Pregnancy Childbirth. 2017 Dec 29;17:444. doi: 10.1186/s12884-017-1631-y (PMC5747179; doi:10.1186/s12884-017-1631-y)
Supplement: Supplementary file 4 — Health centre village meetings summary form. (DOCX 12 kb) [file 12884_2017_1631_MOESM4_ESM.docx]

**Additional file 4. Health centre village meetings summary form**

| Number of villages | Population of 15-49 year age group | Number of women of child bearing age who attended CDTFA meetings | Number of male attendance | Number of health profile records books seen | Number of first trimester attendees identified from the health profile records books | Number of Lead mothers nominated |
| --- | --- | --- | --- | --- | --- | --- |
|  |  |  |  |  |  |  |
